# Pitavastatin Is Anti-Leukemic in a Bone Marrow Microenvironment Model of B-Lineage Acute Lymphoblastic Leukemia

**DOI:** 10.3390/cancers14112681

**Published:** 2022-05-28

**Authors:** Debbie Piktel, Rajesh R. Nair, Stephanie L. Rellick, Werner J. Geldenhuys, Karen H. Martin, Michael D. Craig, Laura F. Gibson

**Affiliations:** 1 Robert C. Byrd Health Sciences Center, West Virginia University Cancer Institute, West Virginia University, Morgantown, WV 26506, USA; dpiktel@hsc.wvu.edu (D.P.); rrnair@gmail.com (R.R.N.); srellick@hsc.wvu.edu (S.L.R.); kamartin@hsc.wvu.edu (K.H.M.); 2 Department of Pharmaceutical Sciences, West Virginia University School of Pharmacy, Morgantown, WV 26506, USA; werner.geldenhuys@hsc.wvu.edu; 3 Department of Microbiology, Immunology and Cell Biology, West Virginia University School of Medicine, Morgantown, WV 26506, USA; 4 Queen’s Health System, Honolulu, HI 96813, USA; micraig@queens.org

**Keywords:** lipid metabolism, drug resistance, metabolism, ALL

## Abstract

**Simple Summary:**

Chemoresistance after chemotherapy is a negative prognostic indicator for B-cell acute lymphoblastic leukemia (ALL), necessitating the search for novel therapies. By growing ALL cells together with bone marrow stromal cells, we developed a chemoresistant ALL model. Using this model, we found that the lipid lowering drug pitavastatin had antileukemic activity in this chemoresistant co-culture model. Our data suggests that pitavastatin may be a novel treatment option for repurposing in chemoresistant, relapse ALL.

**Abstract:**

The lack of complete therapeutic success in the treatment of B-cell acute lymphoblastic leukemia (ALL) has been attributed, in part, to a subset of cells within the bone marrow microenvironment that are drug resistant. Recently, the cholesterol synthesis inhibitor, pitavastatin (PIT), was shown to be active in acute myeloid leukemia, prompting us to evaluate it in our in vitro co-culture model, which supports a chemo-resistant ALL population. We used phospho-protein profiling to evaluate the use of lipid metabolic active compounds in these chemo-resistant cells, due to the up-regulation of multiple active survival signals. In a co-culture with stromal cells, a shift towards anabolic processes occurred, which was further confirmed by assays showing increased lipid content. The treatment of REH leukemia cells with pitavastatin in the co-culture model resulted in significantly higher leukemic cell death than exposure to the standard-of-care chemotherapeutic agent, cytarabine (Ara-C). Our data demonstrates the use of pitavastatin as a possible alternative treatment strategy to improve patient outcomes in chemo-resistant, relapsed ALL.

## 1. Introduction

The detection of minimal residual disease (MRD) after chemotherapy is a negative prognostic indicator in B-cell acute lymphoblastic leukemia (ALL) patients [1]. The presence of MRD is correlated with increased relapsed rates, and monitoring MRD throughout therapy is a standard practice in ALL patients [2]. The site of relapse is also very important for assessing prognosis, with MRD in the bone marrow resulting in an abysmal 15% survival rate compared to extramedullary MRD, where the survival rate approaches 80% [3]. The gene expression profiling of bone marrow samples from patients at diagnosis, and later at relapse, revealed an enrichment of genes in the relapsed patients that were involved in cell death, cell survival, and cell-to-cell signaling [4]. Additionally, bone marrow microenvironment-mediated drug resistance has been shown to be a major factor that contributes to chemo-resistance and MRD in ALL [5,6].

The intensification of the chemotherapy regimen to overcome bone marrow relapse has been relatively unsuccessful in improving therapeutic outcomes [7]. Novel therapies are being developed, with monoclonal antibodies like CD19/CD3-BiTE blinatumomab being approved by the FDA for relapsed/refractory ALL [8]. Similarly, inotuzumab ozogamicin, a CD22-drug conjugated antibody therapy, has resulted in positive outcomes in clinical trials involving patients with relapsed ALL [9]. Furthermore, chimeric antigen receptor T cell therapy for B-ALL has shown a high degree of efficacy in clinical trials [10]. Unfortunately, poor outcomes for relapsed patients with MRD persist, leading to a search for better therapies that aim at targeting the pathways linked to the bone marrow-mediated drug resistance.

Lipid metabolism has gained recent attention as a novel method to target cancers [11], including leukemias [12,13,14]. The resistance to venetoclax with azacytidine was found, in part, to be due to the up-regulation of fatty acid oxidation (FAO) [15] and inhibition of FAO re-sensitized resistant leukemia stem cells [16]. In working towards the goal of delineating the biological pathways of bone marrow-mediated drug resistance in B-cell ALL [17], our lab has previously described and validated a co-culture model that mimics the in vivo leukemic bone marrow microenvironment [18]. Using this model, we have shown that the bone marrow niche regulates not only the survival of leukemic cells, but also supports a stem cell-like leukemic population that is drug resistant [19,20]. We developed a procedure for extracting drug-resistant leukemic cells from this model, which we refer to as phase dim (PD) cells; these migrate beneath the adherent bone marrow stromal (BMSC) or osteoblast (HOB) cells of the co-culture [21]. The characterization of the PD cells demonstrated that they are dormant (G_0_ cell cycle), have altered metabolism, and have a multi-drug resistant phenotype, all of which are hallmarks of MRD [22]. Interestingly, the quiescence observed in PD cells within the context of the bone marrow niche was lost when the cells were released from the niche. They proliferated robustly, mimicking disease relapse [22]. An understanding of the molecular pathways involved in chemo-resistance will allow for the identification of targets for therapies aimed at this clinically challenging subset of tumor cells.

Recently, a clinical trial was established to target lipid metabolism in acute myeloid leukemias (AML) using the HMG-CoA-reductase inhibitor, pitavastatin. Pitavastatin is a lipid-lowering drug that is currently in a clinical trial with venetoclax as a therapeutic option for the treatment of AML and chronic lymphocytic leukemia (NCT04512105). Statins, including pitavastatin, have been explored in several cancers. Pitavastatin holds much promise as a therapeutic due to its longer half-life in comparison to atorvastatin or simvastatin, and studies have reported it to be synergistic when combined with standard-of-care agents [23,24,25,26,27,28]. In light of these reports, the present study was undertaken to evaluate the pharmacology of pitavastatin, and other metabolic inhibitors, on a drug-resistant leukemic cell population in an in vitro co-culture model, mimicking the protection afforded by the bone marrow niche microenvironment [17]. Targeting the drug-resistant PD population through the inhibition of these metabolic survival signals will shed light on alternative treatment modalities to be used in combination therapy with current standard-of-care agents to improve patient outcomes.

## 2. Materials and Methods

### 2.1. Cell Culture and Materials

REH human ALL cells (ATCC #CRL-8286) were purchased and maintained in RPMI 1640 supplemented with 10% FBS and 1x streptomycin/penicillin antibiotics. TOM-1 (DSMZ ACC#578) and SUP-B15 ALL cells (ATCC #CRL-1929) were purchased and maintained in RPMI 1640 supplemented with 10% FBS, 0.05 mM β-mercaptoethanol, and 1x streptomycin/penicillin antibiotics. Nalm27 cells were obtained from the Fujisaki Cancer Center and maintained in RPMI 1640 supplemented with 10% FBS, 0.05 mM β-mercaptoethanol, and 1x streptomycin/penicillin antibiotics. Human osteoblasts (HOB) were purchased from PromoCell (Cat No: C-12720, Heidelberg, Germany) and cultured according to the vendor’s recommendations. De-identified primary bone marrow stromal cells (BMSC) and AML cells were provided by the WVU Cancer Institute Biospecimen Processing Core and the WVU Department of Pathology Tissue Bank. ALL cell lines were authenticated by short tandem repeat (STR) analysis (University of Arizona Genetics Core, Tucson, AZ, USA) and maintained in 6% CO_2_ in normoxia at 37 °C.

### 2.2. Co-Culture and Isolation of Leukemic Cell Populations

Co-culture conditions were followed as previously described [21]. Briefly, 1 × 10^6^ ALL cells were seeded on an 85% confluent BMSC or HOB layer and maintained in 5% O_2_. The co-culture was fed every 4 days, and treatments were added based on individual experiments (detailed below). On the 12th day in culture, the ALL cells were isolated for further processing. The leukemic cell population that was in suspension and not interacting with the stromal cells was collected and designated as suspended cells (S). The leukemic cells that were buried under the BMSC were separated by size exclusion with Sephadex G-10 after vigorous washing to remove all leukemic cells adhered to the top of the BMSC or HOB [21]. Buried leukemic cells were designated phase dim cells (PD) and have been previously described to be the most chemo-resistant population [19,20,29,30]. As such, they are not assumed to be identical, but rather are used as a model for refractory tumor cells that are known to be clinically problematic in the treatment of ALL. The S and PD cells from the co-culture were compared to ALL cells grown in media alone (M).

### 2.3. Kinase Array Profiling

REH cells were either left untreated or were treated with 0.5 μM MK-2206 (Selleckchem, Houston, TX, USA) for 2 days. Following isolation, the cells were lysed, and protein was isolated, quantitated, and subjected to the Proteome Profiler Human Phospho-Kinase Array Kit according to the manufacturer’s instructions (R&D Systems, Minneapolis, MN, USA). The density of the signals was analyzed using Amersham Imager 600 software. The pixel densities were then used to measure the fold-change in phosphorylation levels of the protein.

### 2.4. Lipid Synthesis Inhibition

REH cells were treated for 2 days with an ATP citrate lyase inhibitor, BMS-303141 (10 μM) (Tocris, Minneapolis, MN, USA), an HMG-CoA reductase inhibitor, pitavastatin (1 μM) (Tocris, Minneapolis, MN, USA), a chemotherapy agent, cytarabine (Ara-C, 0.1 μM) (Sigma Aldrich, St. Louis, MO, USA) or the combinations, as indicated. Following treatment, the media cells (M), suspended cells (S), and phase dim cells (PD) were recovered, and the viability and total cell count were analyzed using the trypan blue exclusion method.

### 2.5. AKT Inhibition

REH cells were treated with cytarabine (Ara-C, 1 μM) (Sigma Aldrich, St. Louis, MO, USA) or MK-2206 (0.5 μM), or a combination of both for 2 days. Following treatment, the suspended cells (S) and the phase dim (PD) cells were recovered, and viability was analyzed using the trypan blue dye exclusion method.

### 2.6. Real-Time PCR Analysis

REH or TOM-1 cells were isolated and processed for RNA; real-time RT-PCR was performed using GLUT-1, 3 and 4 primers (Integrated DNA Technologies): GLUT-1, 5′-GATTGGCTCCTTCTCTGTGG-3′ and 5′-TCAAAGGACTTGCCCAGTTT; GLUT-3, 5′-CAGGCACACGGGCAGATAG-3′ and 5′-GCAGGCTCGATGCTGTTCAT-3′; GLUT-4, 5′-CTGCCAGAAAGAGTCTGAAGC-3′ and 5′-CCTTCAGCTCAGCCAGCACT-3′. β-actin primers (RealTimePrimers.com, accessed on 5 May 2022) were used to normalize the data: ACTB, 5′-CTGGAACGGTGAAGGTGACA-3′ and 5′-AAGGGACTTCCTGTAACAATGCA-3′.

### 2.7. Glycogen Assay

REH cells (2 × 10^6^) from each cell population were used to measure the glycogen content using a glycogen colorimetric assay kit according to the manufacturer’s instructions (Biovision, Milpitas, CA, USA).

### 2.8. Cell Proliferation Assay

Cells were plated in 96-well flat bottom clear plates at a density of 50,000 cells per well. Cells were treated with BMS-303141 or pitavastatin at indicated concentrations, and the cell proliferation was tested after 3 days of incubation with the compounds; and 48 h for the primary AML patient samples. A cell counting kit was utilized according to the manufacturer’s instructions (Dojindo Molecular Technologies Inc., Rockville, MD, USA, Cat # CK04). Briefly, 10 μL of the assay reagent was added to each well and incubated for 2 h at 37 °C, after which the plates were read on a BioTek Cynergy 5 plate reader at 450 nm absorbance. Untreated cells were used as controls.

### 2.9. Western Blot Analysis

The isolated cells were lysed using RIPA buffer, and the resultant protein concentrations were determined using the bicinchoninic acid (BCA) protein assay kit (Pierce-Thermo Fisher Scientific, Waltham, MA, USA). Equal quantities of proteins were resolved on SDS-PAGE gels and transferred to nitrocellulose membranes. Membranes were blocked in TBS with 5% nonfat dry milk and 0.1% Tween 20 and probed with the indicated primary antibodies. AMPK and phospho-AMPK antibodies, lipin-1, ATP citrate lyase (ATP-CL) and phospho-ATP-CL, and acetyl-CoA carboxylase (ACC) and phospho-ACC were all purchased from Cell Signaling Technology (Danvers, MA, USA). After incubation with horseradish peroxidase-conjugated secondary antibodies, signal was visualized using Amersham ECL Prime Western blot detection reagent (GE Life Technologies, Boston, MA). Finally, densitometry and normalization were completed. Western blots are representative of at least three independent experiments.

### 2.10. Flow Cytometry Analysis

The isolated ALL cell populations were treated with either pan-GLUT inhibitor (5 μm) (GLUTi II, Calbiochem Cat# 400035) or DMSO for 4 h and then incubated in media containing 50 μM 2-NBDG (Life Technologies, Carlsbad, CA, USA), a fluorescent glucose analog. After 30 min incubation, the cells were subjected to flow cytometry (BD LSRFortessa) to measure glucose content in cells.

### 2.11. Nile Red Staining

Following isolation, 4 × 10^6^ cells were re-suspended in PBS containing 2 μM of Nile Red (Invitrogen, Carlsbad, CA, USA) for 10 min. The cells were rinsed with PBS, re-suspended in PBS, and cytospun onto slides. Nile Red staining was visualized using a Zeiss LSM 510 confocal microscope, exciting with the 488 nm laser and collecting with a LP 615 emission filter.

### 2.12. Statistical Analysis

All experiments were carried out independently and repeated at least three times, unless otherwise noted. Student’s t-test (un-paired) was utilized to compare between two groups. For comparison between treatment groups of three or more, we first performed one-way ANOVA followed by a post-hoc Tukey’s test. The data are represented as mean ± SEM and statistical difference was noted as significant if *p* < 0.05.

## 3. Results

### 3.1. Drug-Resistant Phase Dim (PD) Cells Have Activated Survival Signals That Are Not Abrogated by Targeted Therapy

**Figure 1:**
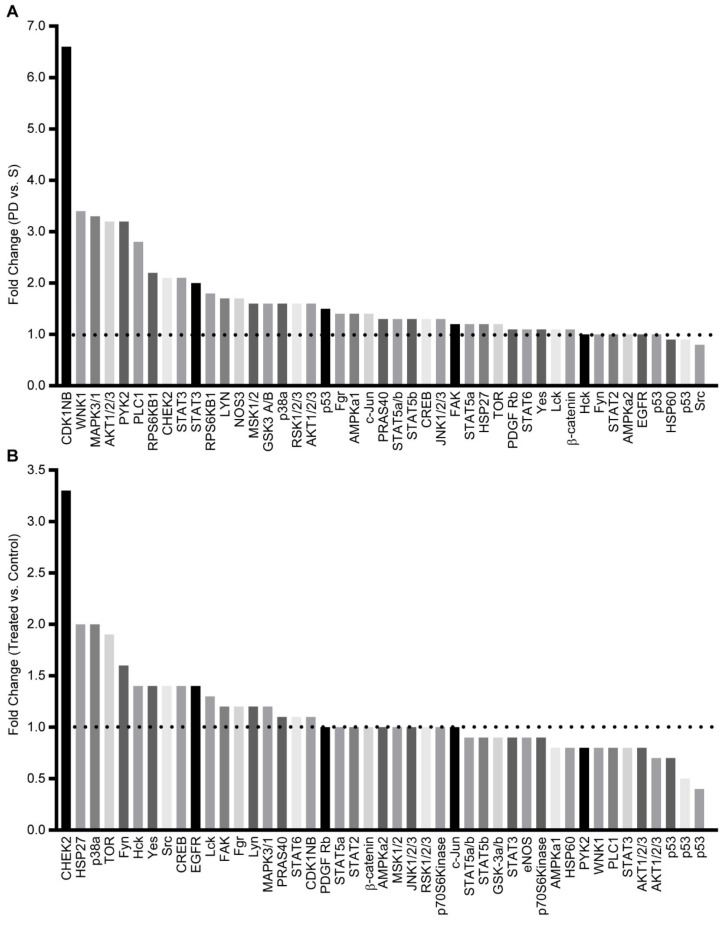
Activation of survival signaling pathways in drug-resistant phase dim (PD) cells. (**A**) REH cells were co-cultured with BMSC, the S and PD ALL cells were isolated, and the phosphorylation of protein kinases was compared with the PD expression level compared to the S sub-population. (**B**) REH cells were co-cultured with BMSC and left untreated (control) or treated with MK-2206. The PD REH tumor cells cultures were isolated from the control and MK-2206 cultures, and the relative level of protein kinase phosphorylation was determined.

Previously, we reported that a co-culture of leukemia cells with BMSC or HOB supports a population of Ara-C resistant cells [19,20]. In an effort to characterize specific contributors to this chemo-resistant phenotype, potential therapeutic targets in survival signaling pathways were identified by kinase array profiling. Initial studies were done with REH leukemic cells co-cultured with bone marrow stromal cells (BMSCs), and the phase dim (PD) and suspension (S) leukemic cells were isolated. PD cells had increased phosphorylation of several survival signaling proteins, including AKT (Figure 1A). To determine whether targeted AKT inhibition would abrogate the survival signaling pathways and allow the PD cells to become sensitive to chemotherapy, co-cultures were treated with a pan-AKT inhibitor (MK-2206). We observed that in response to MK-2206, AKT phosphorylation decreased, whereas the phosphorylation of other proteins, such as CHEK2, a regulator of cell division, and HSP27, which can inhibit apoptosis, increased (Figure 1B). Additionally, when the co-cultures were treated with MK-2206 (500 nM) in combination with Ara-C, a chemotherapy standard-of-care agent used in the treatment of leukemia, there was no significant decrease in cell viability, and, in fact, the AKT inhibitor reversed the sensitivity of the PD cells to Ara-C (Figure S1). This suggests that inhibition of AKT signaling may lead to the activation of other compensatory survival signaling pathways and serves as one explanation for the ineffectiveness of targeted single inhibition (Figure S1).

### 3.2. Phase Dim (PD) Cells Have Characteristics of Anabolic Metabolism

Within the co-culture, the PD cells are buried underneath the adherent cell layer, having decreased access to nutrients in comparison to the cells in suspension. Due to the decreased nutrients and increased energy demands caused by rapid proliferation, it is common for tumor cells to undergo the “Warburg effect,” utilizing glycolysis as their primary energy source even in the presence of oxygen. We posited that the PD cells in the niche environment would have increased expression of proteins associated with nutrient uptake related to the AMPK signaling pathway, as well as the storage of lipids, based on recent findings with other leukemia agents [13].

**Figure 2:**
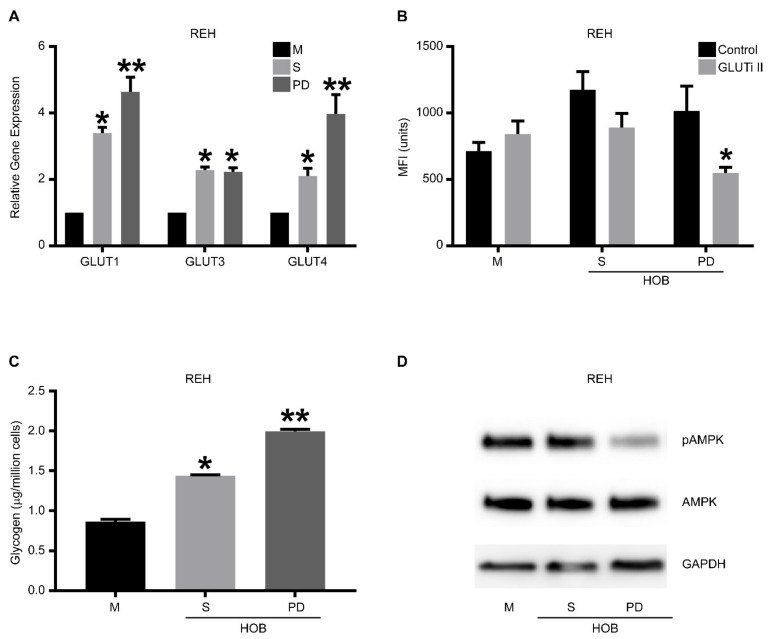
Drug-resistant phase dim (PD) cells are characterized by anabolic metabolism. REH cells were co-cultured with HOB, and the tumor cells in suspension (S) and the phase dim (PD) cells were isolated and compared to REH cells grown in media alone (M). (**A**) RT-PCR was used to determine the expression levels of GLUT-1, 3, and 4. Data is represented as mean ± SEM and is a representative of an experiment performed in triplicate at least two independent times. (**B**) The different cell populations were treated with a pan GLUT inhibitor (GLUTi II) or vehicle control followed by incubation with 2-NBDG. Flow cytometry was used to measure glucose content in cells shown as mean fluorescent intensity (MFI). (**C**) A glycogen colorimetric assay was used to measure the glycogen content in the isolated tumor cell populations. (**D**) Western blot analysis was used to measure the amount of AMPK and phospho-AMPK. The blots are representative of three independent experiments. * *p* < 0.05 when compared to cells grown in media alone. ** *p* < 0.05 when compared to suspension cells isolated from co-culture or tumor cells grown in media alone. The uncropped blots are shown in Supplementary Materials.

Previous reports have suggested that altered metabolism in cancer, especially changes in glucose and lipid metabolism, can induce altered drug sensitivity and resistance patterns [14,31,32]. Therefore, we first evaluated our REH cells for the expression of glucose transporters and found that REH cells in a co-culture (S and PD) have a significantly increased expression of GLUT 1, 3, and 4 compared to REH cells grown in media alone (Figure 2A). We then inhibited glucose transport in the REH cells under the same culture conditions using the GLUTi-II inhibitor and measured the uptake of glucose with 2-NBDG, a fluorescent glucose analog. Unique to the PD cells was the significantly reduced glucose uptake following the inhibition of glucose transporters (Figure 2B), suggesting that these PD cells have an increased glucose utilization need. We replicated these experiments using the SUP-B15 and TOM-1 cell lines and determined that, consistent with REH cells, there was an increased expression of glucose transporters in cells grown in co-culture (Figure S2A), and the PD cell population had significantly less 2-NBDG uptake when glucose transporters were inhibited (Figure S2B). Furthermore, using the pan-GLUTi II inhibitor (2.5 and 5 μM), we found a significant decrease in the number of viable cells in both suspension (S) and PD cells in co-culture (Figure S3), as would be expected from cells which survive in a bone niche environment. We also measured the glycogen storage in REH cells grown in media alone or co-culture with either HOB (Figure 2C) or BMSC (Figure S2C), and we determined that REH cells in co-culture had increased glycogen storage, with PD cells having significantly higher glycogen storage than cells in suspension. Finally, we evaluated AMPK expression and activation in REH and SUP-B15 cells, as AMPK is a master regulator of cell metabolism. Both REH (Figure 2D) and SUP-B15 PD cells (Figure S2D) had decreased phosphorylation of AMPK, consistent with our observations of increased glycogen storage and an anabolic phenotype.

### 3.3. Drug-resistant PD Cells Have Increased Lipid Content

**Figure 3:**
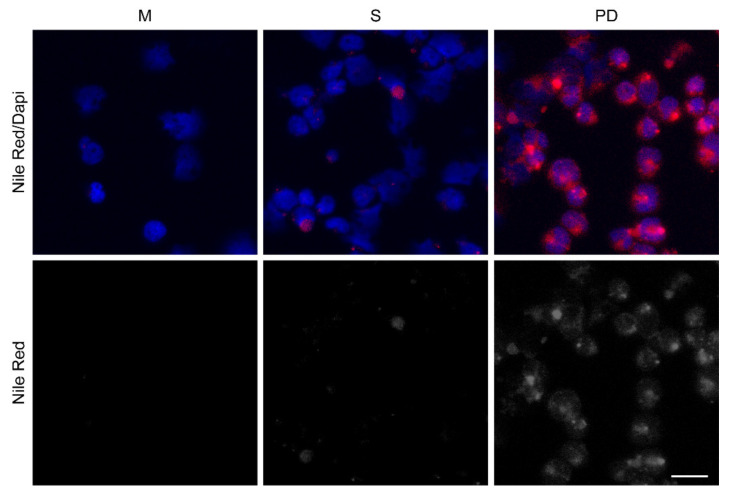
Drug-resistant phase dim (PD) cells have increased lipid content. REH cells were co-cultured with (HOB), and the cells in suspension (S) and the phase dim (PD) cells were isolated and compared to REH cells grown in media alone (M). Nile Red staining was completed to visualize lipid content. Scale bar = 20 μm. Red (Nile Red); Blue (DAPI nuclear stain).

To further characterize an anabolic phenotype in the tumor cells in the bone microenvironment, we co-cultured REH or TOM-1 cells with HOBs, as a representative cell type to mimic the bone marrow microenvironment, and subsequently stained the isolated leukemic cells with Nile Red to evaluate lipid content. The PD cells had a higher lipid content, suggesting increased lipid synthesis and/ or storage (Figure 3 and Figure S4). To further interrogate the mechanism behind the increased lipid content, the proteins associated with lipid regulation (ATP-citrate lyase (ATP-CL), acetyl-CoA carboxylase (ACC), and lipin-1), were evaluated by Western blot analysis to determine the expression levels of these proteins in our tumor cells. These experiments were completed in three ALL cell lines (Nalm-27; Ph+), REH, and TOM-1; Ph+) and cells were grown in either media alone or in co-culture with HOB or BMSC. Consistent with our Nile Red staining, the PD cells had increased levels of phosphorylated ATP-CL, with minimal changes in ACC or lipin-1, compared to cells grown in media alone (Figure S5).

### 3.4. Tumor Cell Viability Decreases When ALL Cells Are Treated with Inhibitors of Lipid Synthesis

**Figure 4:**
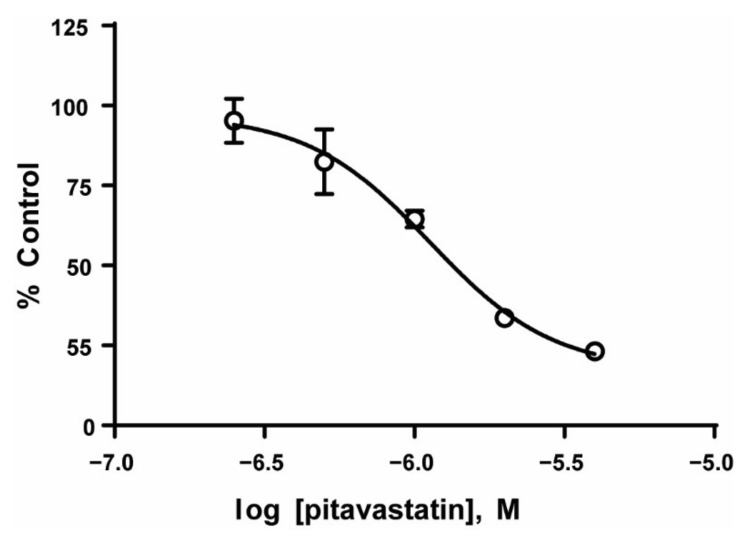
Inhibition of lipid synthesis decreases ALL cell proliferation. REH cell lines were treated with the indicated concentrations of pitavastatin, and the cell proliferation was measured as described in the methods. The data are represented as mean ± SD of a study performed in triplicate at least three independent times.

**Figure 5:**
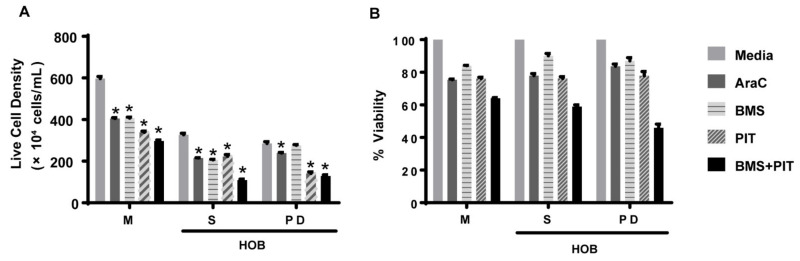
Pitavastatin decreases ALL cell viability in co-culture. REH cells were grown in a co-culture with HOB and were treated with Ara-C (0.1 μM), BMS-303141 (BMS; 10 μM), pitavastatin (PIT; 1 μM) or a combination of BMS-141303 and pitavastatin (BMS + PIT). The cells in suspension (S) and the phase dim (PD) cells were isolated and compared to REH cells grown in media alone (M). The total cells and the live cell population (**A**) were used to calculate the % viability (**B**). The data is presented as mean ± SEM and is representative of a study performed in triplicate and conducted three independent times. * *p* < 0.05 when compared to vehicle/media treated.

Based on the data showing increased lipid synthesis in the PD cells, it is plausible to propose that the tumor cells in the co-culture model, which mimics the bone niche microenvironment, may utilize lipids as an alternative energy source, and that lipid synthesis/metabolism may contribute, in part, to tumor cell chemotherapy resistance. To determine whether the lipid metabolism could be a potential therapeutic target [32,33], we first exposed REH and TOM-1 cells, cultured in media alone, to a range of concentrations of pitavastatin or BMS-303141, which inhibit cholesterol synthesis and ATP-CL, respectively, and are used as an alternative control of lipid metabolism. We observed a concentration-dependent decrease in cell proliferation, with pitavastatin having an IC_50_ of 1.12 µM (REH) (Figure 4). We then expanded our investigation of the inhibitors to our co-culture model and determined that the PD cells, although not sensitive to BMS-303141 alone, were sensitive to the combination of BMS-303141 and pitavastatin, (Figure 5A,B). The synergistic or additive effect of pitavastatin with BMS-303141 suggests that the divergent pathways are compensating when treated with individual compounds, but the combination is able to attenuate this compensation, supporting a premise that cells in the bone microenvironment survive by multiple independent, compensatory metabolic pathways. These data further suggest that the ability of the tumor cells to synthesize lipids and have an additional “energy reserve” may be critical to their survival and subsequent resistance to chemotherapeutic agents when residing and surviving in the bone marrow niche environment.

## 4. Discussion

A dysregulated lipid metabolism is implicated in contributing to resistance in several cancers, with similar effects seen in leukemias [16]. Clinically, retrospective studies have demonstrated that MRD leads to relapse in ALL patients, and most often, relapse is accompanied by a resistance to standard-of-care therapies [13,14,17]. Recently, a clinical trial was initiated on the lipid-lowering drug, pitavastatin, in AML. Based on this observation, as well as recent studies which implicated lipid metabolic changes with MRD or drug resistance, the present study was initiated to evaluate the effect of pitavastatin on the lipid metabolism of a bone marrow niche co-culture model that mimics B-cell ALL MRD in vitro [34]. We observed that the inhibition of lipid synthesis using a fatty acid synthesis inhibitor (BMS-303141) and a cholesterol synthesis inhibitor (pitavastatin) leads to cell death in the PD leukemic cells, which are resistant to Ara-C-induced cell death in our co-culture model. Our data also suggest that pitavastatin is potentially more potent in B-cell ALL as compared to human AML (Figure S6). Concurrent with overlapping signaling pathways is the switch to anabolism in the bone microenvironment, characterized by an increase in glycogen storage and simultaneous shut down of AMPK activity. Furthermore, also consistent with the decrease in AMPK activity is our observation that the PD cells, which have a dormant, drug-resistant phenotype, have increased lipid storage.

We previously reported a pivotal role for activated AKT in protecting B-cell ALL cells against chemotherapy-mediated cell death [35]. Consistent with our earlier report, AKT was one of the kinases that was significantly phosphorylated in PD tumor cells. Of note, AKT activation is also frequently elevated in ALL patients and is correlated with a poor response to chemotherapy [36,37]. Based on its previously documented role, we tested whether the targeted inhibition of AKT was sufficient to eradicate the PD cells in our co-culture model by using a pan-AKT inhibitor, MK-2206, which has been shown to have clinical benefit in cancer patients [38]. Our study suggests inherent plasticity in the signaling profile of PD leukemic cells that are resistant to chemotherapy in our co-culture model and may represent the leukemic cells that are able to evade chemotherapy and contribute to relapse/ chemo-resistant disease in patients. Combinations of traditional therapies and targeted therapies against critical survival signaling proteins, although very attractive and effective at reducing bulk tumor populations, may result in evolutionary pressure on leukemic cells in protective bone marrow niches, leading to the clonal outgrowth of a multi-drug resistant phenotype.

Both dormancy and a drug resistant phenotype are known to co-exist in MRD cells in ALL [39]. The PD cells in our co-culture model show an increased activation of CDKN1B, which correlates with previous data from our lab showing that the PD cells are also in G_0_ cell cycle arrest, demonstrating that our co-culture model, and studies utilizing the PD cell population, are relatable to leukemic cells that contribute to MRD in patients [29]. In the present study, the inhibition of AKT by MK-2206 does decrease the levels of activated CDKN1B, but similar to the survival signaling pathways, the cells compensate by increasing the levels of activated CHEK2, which is a known regulator of cell division.

Active AKT signaling can lead to increased glucose uptake via an up-regulation of glucose transporter (GLUT) expression in cancer cells [40,41]. Moreover, GLUT family members, especially GLUT1, play a pivotal role in the anabolic metabolism and drug resistance in ALL cells [41,42]. We have demonstrated that the chemo-resistant PD cell population has increased transcript levels for GLUT-1, 3, and 4. More importantly, the inhibition of GLUT activity significantly decreased the uptake of glucose, predominantly in the drug-resistant PD cells, demonstrating the influence of the co-culture microenvironment on the leukemic cells.

Glycogen is the storage form of glucose, and it has been shown to directly bind to, and inhibit, the energy stress sensor AMPK [43,44,45]. We showed that increased glucose uptake resulted in an increased glycogen content within the drug-resistant PD cells. Furthermore, this increase in glycogen content correlated with the significant inhibition of AMPK activity in the drug-resistant PD ALL cell population. AMPK is a cellular energy sensor that is activated when there is a decrease in ATP levels, resulting in an inhibition of anabolism and increased glycolysis and fatty acid oxidation [46]. Specifically, activated AMPK phosphorylates and inactivates acetyl-CoA carboxylase (ACC) and 3-hydroxy-3-methylglutaryl-CoA reductase (HMGR), the regulators of fatty acid and sterol synthesis, respectively [47,48]. The end result of this activation is the depletion of lipid stores within the cells, caused by the increased uptake and subsequent oxidation of lipids within the mitochondria.

Lipids form an important structural backbone of cells, as well as specialized structures, like lipid rafts, that are important in the assembly of cell surface receptors and signaling complexes on the membranes [49]. Indeed, the use of statins, a potent HMGR inhibitor, has been widely reported to induce cell death in various cancer cells, including ALL [50]. However, epidemiological studies have not shown a clear decrease in cancer incidence in patients [51]. One reason for this discrepancy is the need for very high doses of statins to induce death in cancer cells in vitro. At the same time, ACC inhibitors have been tested and shown to decrease de novo lipogenesis and induce death in cancer cells [52]. Unfortunately, the effect of decreased lipid synthesis through the inhibition of the ACC pathway was diminished by the subsequent engagement of acetate metabolism pathways that compensated for the lipid turnover deficiency [33]. To overcome both of these disadvantages, we used a combination of pitavastatin (HMGR inhibitor) and BMS-303141 (ACC inhibitor) as a potential therapeutic strategy to induce cell death in the resistant PD cells. We demonstrated that these inhibitors are synergistic, and the concentration of each inhibitory molecule, when used in combination, is significantly lower compared to the concentration necessary to induce death in resistant cells when each inhibitor is used individually.

## 5. Conclusions

Overall, our findings have identified an anabolic phenotype in ALL cells that provides energy reserves in the form of stored glycogen and lipids. Previously, we reported that in our co-culture model, the PD cell population is resistant to Ara-C-induced cell death. The data presented in the current report suggest that treatment with pitavastatin leads to decreased viability in the chemo-resistant PD cell population. This suggests that lipid synthesis may be a critical component of ALL tumor cell survival, even in sanctuary sites like the bone marrow microenvironment. Therapies targeting these anabolic characteristics may help eradicate MRD or chemo-resistant populations in B-cell ALL patients, and as such, may underpin novel treatment strategies.

## Data Availability

Data are available upon request to the corresponding author.

## Supplementary Materials

The following supporting information can be downloaded at: https://www.mdpi.com/article/10.3390/cancers14112681/s1, Figure S1: REH cells in co-culture are resistant to AKT inhibition by MK-2206; Figure S2: ALL cells are characterized by anabolic metabolism in co-culture; Figure S3: Metabolic targeted GLUTi II inhibitor decreases viability in ALL cells in co-culture; Figure S4: Drug-resistant phase dim (PD) cells have increased lipid content; Figure S5: The fatty acid synthesis pathway is activated in ALL cells; Figure S6: AML cells treated with pitavastatin.
