# Supplementary material for: Pitavastatin Is Anti-Leukemic in a Bone Marrow Microenvironment Model of B-Lineage Acute Lymphoblastic Leukemia
Source: Cancers (Basel). 2022 May 28;14(11):2681. doi: 10.3390/cancers14112681 (PMC9179467; doi:10.3390/cancers14112681)
Supplement: Supplementary file 1 [file cancers-14-02681-s001.zip › cancers-1703872-original-images.pdf]

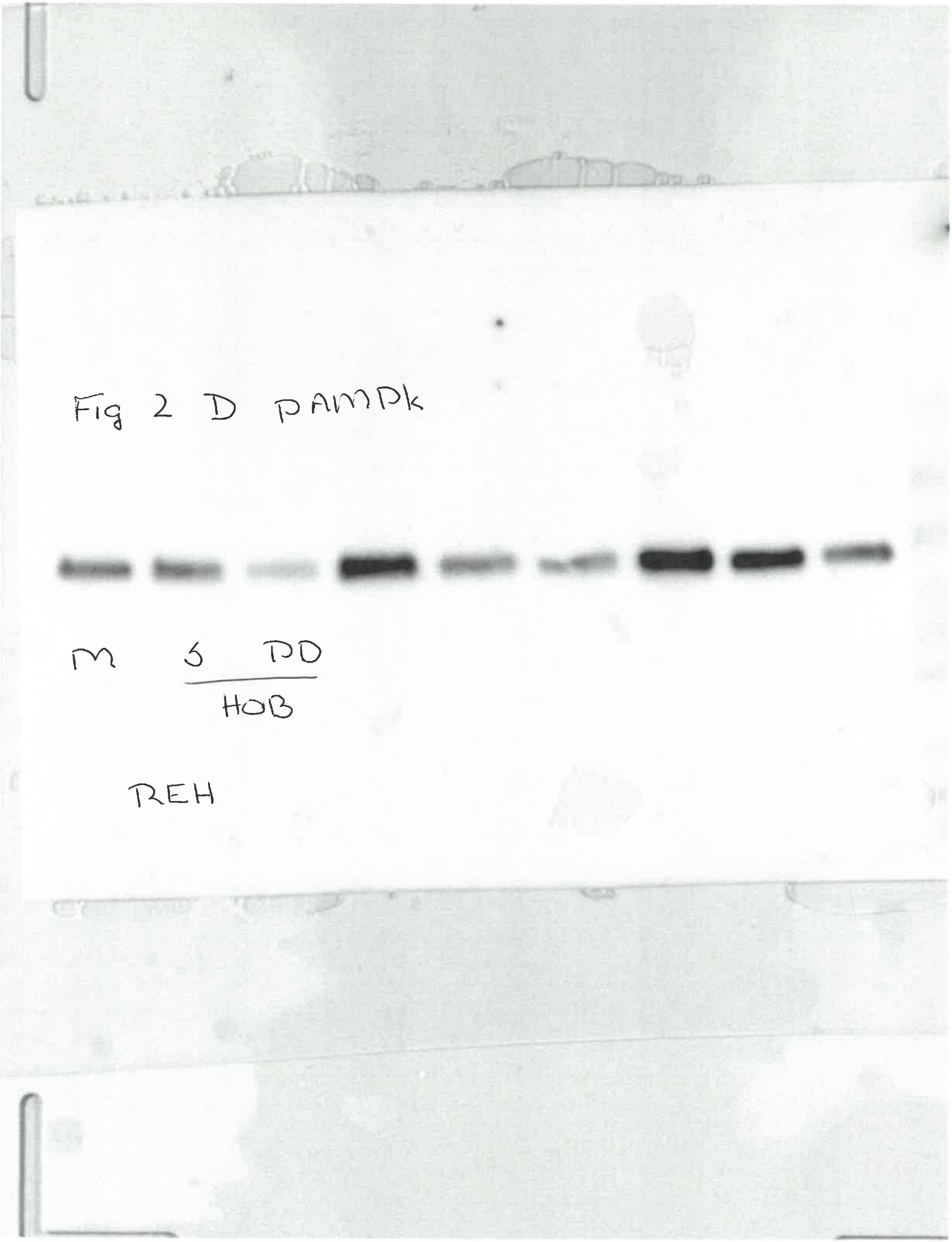
Fig 2 D pAMPk
M S PD
HOB
REH

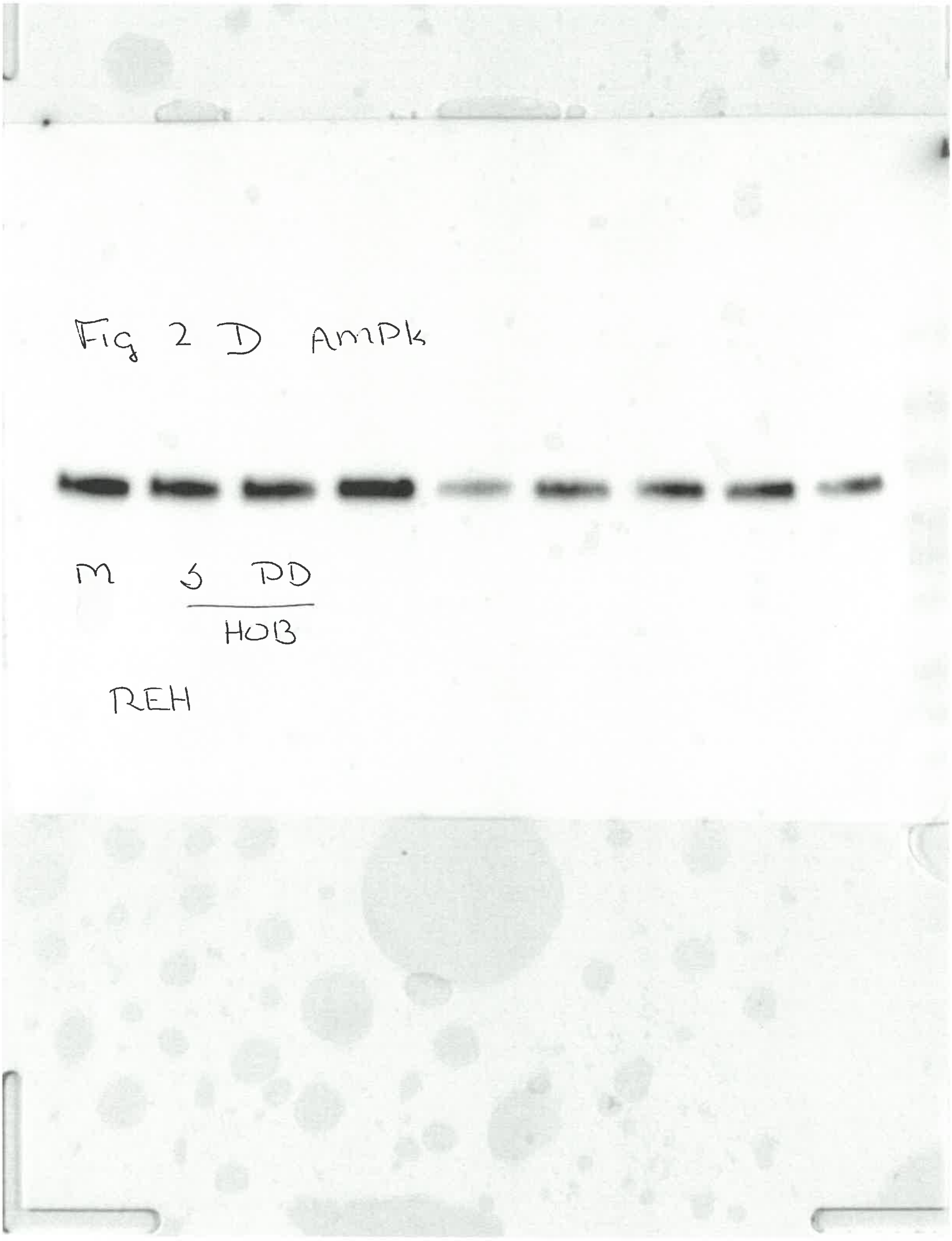
Fig 2 D AMPK
M
S PD
HOB
REH

Fig 2 GAPDH

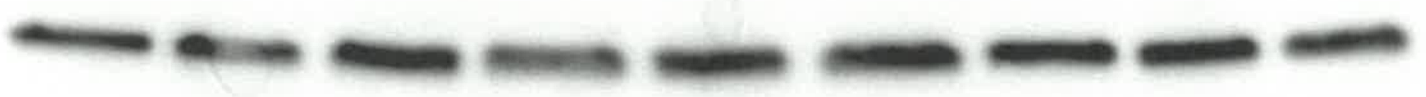

M S PD

HOB

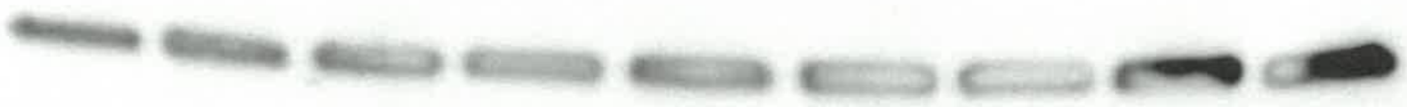

pAMPk Fig S2 D

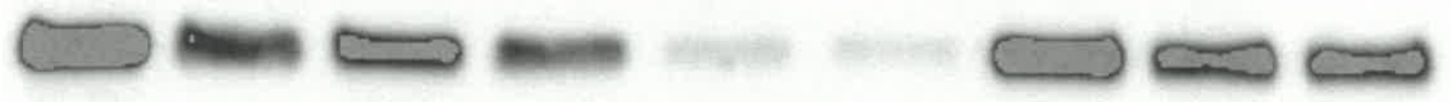

M S PD

HOB

4

AMPk Fig S2 D

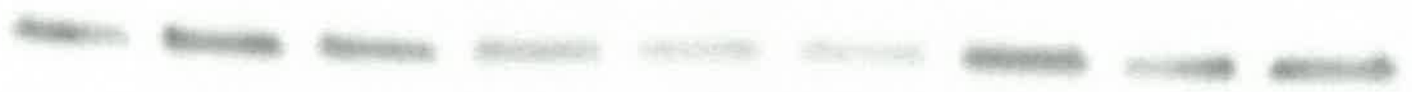

M S PD

HOB

Sup B15

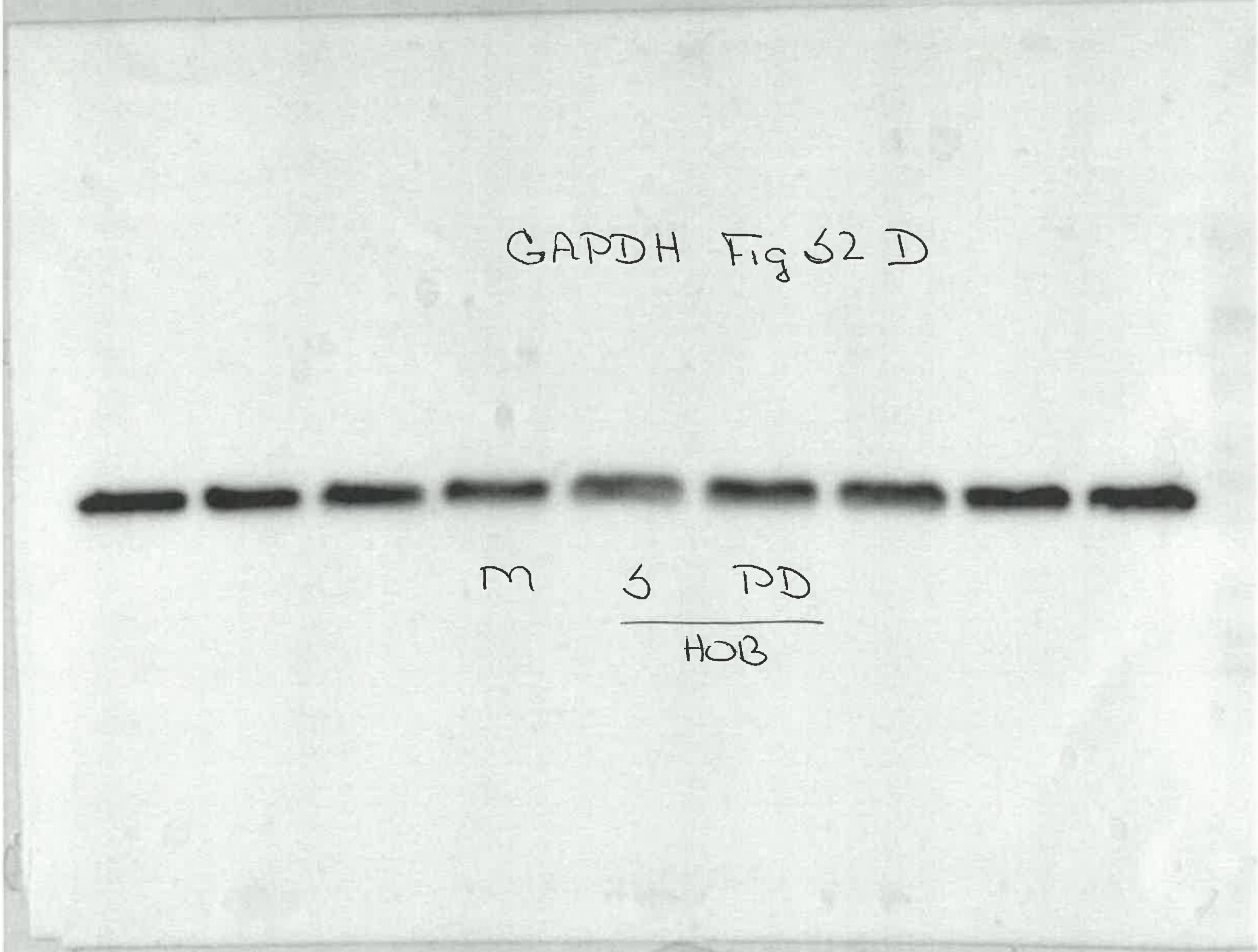
GAPDH Fig S2 D
M S PD
HOB

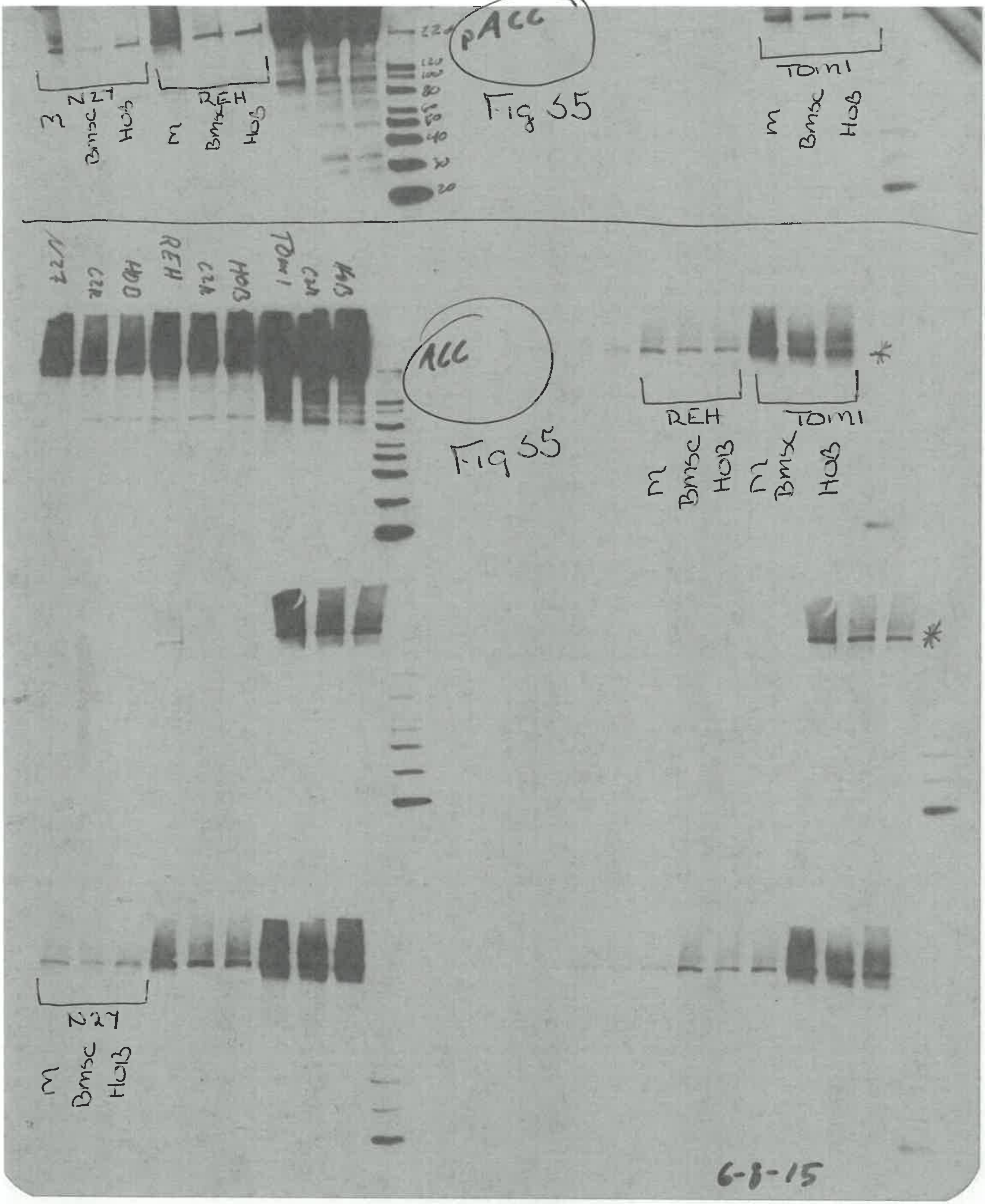
pACC
Fig S5
Z27
m
Bmsc
HOB
REH
m
Bmsc
HOB
TOM1
m
Bmsc
HOB
220
120
100
80
60
50
40
30
20
ACC
Fig S5
REH
m
Bmsc
HOB
TOM1
m
Bmsc
HOB
Z27
m
Bmsc
HOB
6-8-15

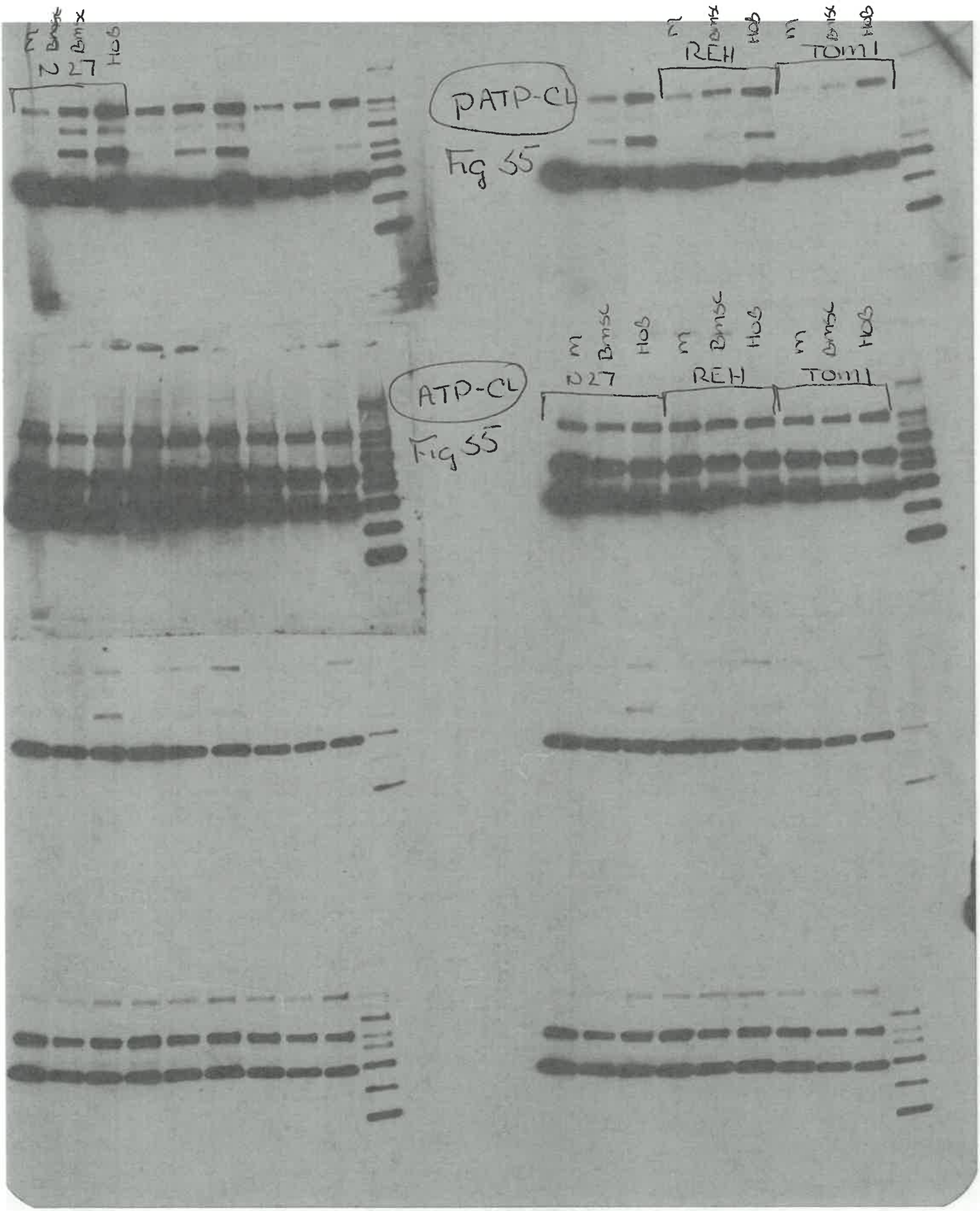

PATP-CL
Fig 55
REH
TOM1
ATP-CL
Fig 55
M
Bmsc
HOB
N27
REH
TOM1

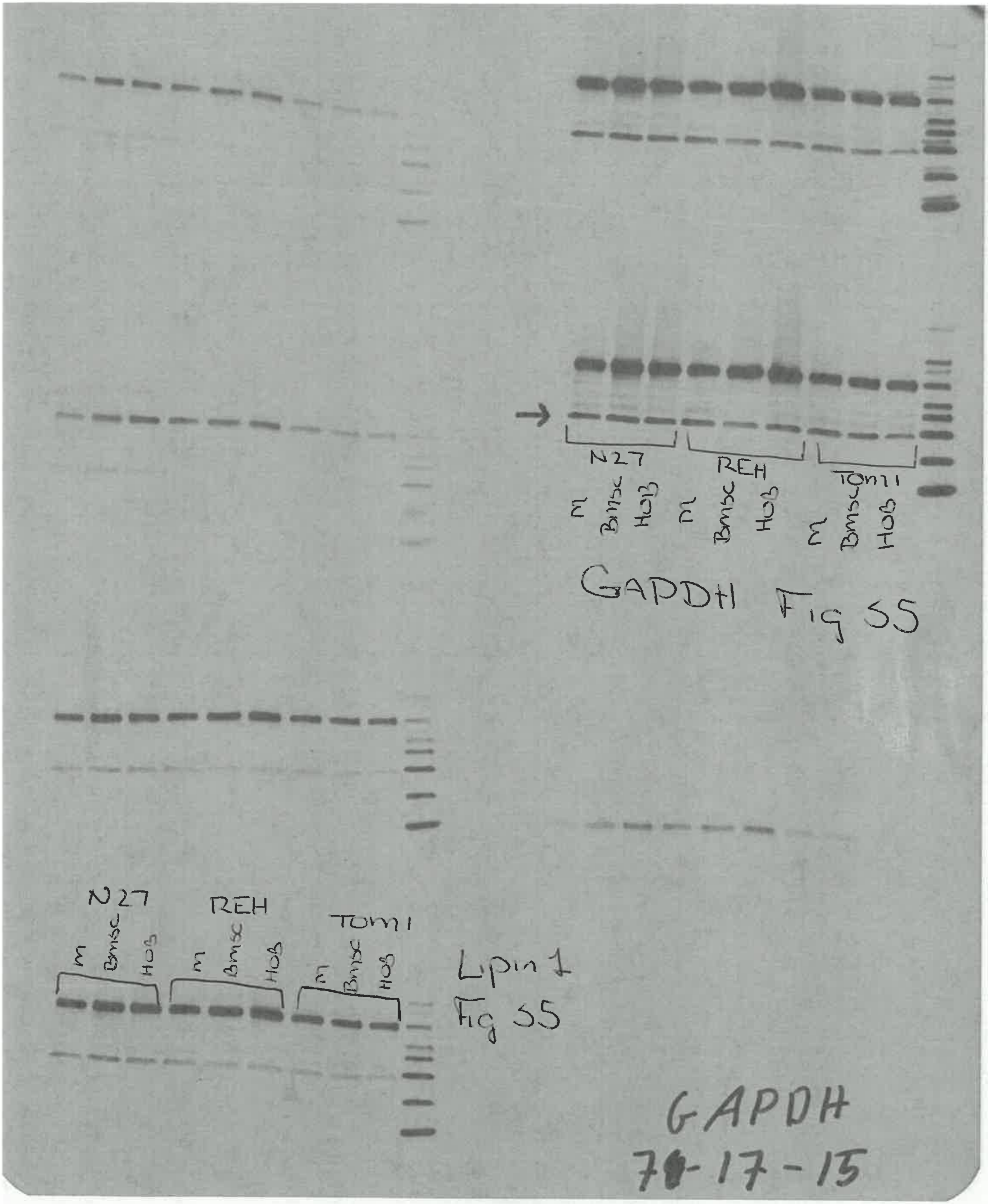
N27
REH
Tom1
M
Bmsc
HOB
GAPDH Fig S5
Lipin 1
Fig S5
GAPDH
