# Supplementary material for: Pitavastatin Is Anti-Leukemic in a Bone Marrow Microenvironment Model of B-Lineage Acute Lymphoblastic Leukemia
Source: Cancers (Basel). 2022 May 28;14(11):2681. doi: 10.3390/cancers14112681 (PMC9179467; doi:10.3390/cancers14112681)
Supplement: Supplementary file 1 [file cancers-14-02681-s001.zip › cancers-1703872-supplementary.pdf]

Supplementary Data

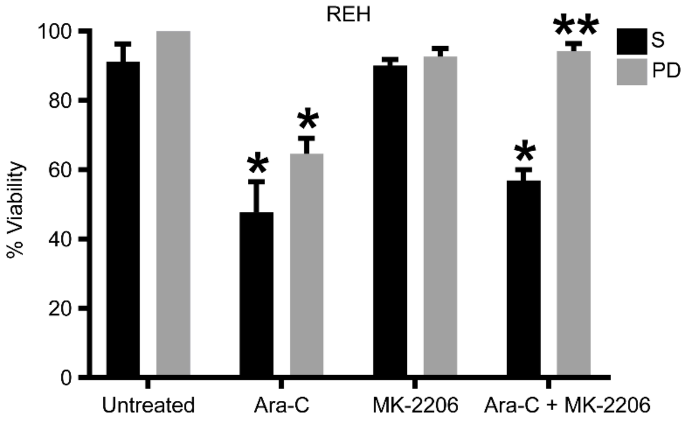

Figure S1. REH cells in co-culture are resistant to AKT inhibition by MK-2206. REH cells were co-cultured with BMSC and were treated with either Ara-C (1 μM) or MK-2206 (0.5 μM) or a combination of both for 48 hrs. At the end of the treatment, the suspended cells (S) and the phase dim (PD) cells were isolated, and the viability was analyzed using the trypan blue dye exclusion method. * $p<0.05$ when compared to S or PD untreated cells. ** $p<0.05$ when compared to PD cells treated with Ara-C.

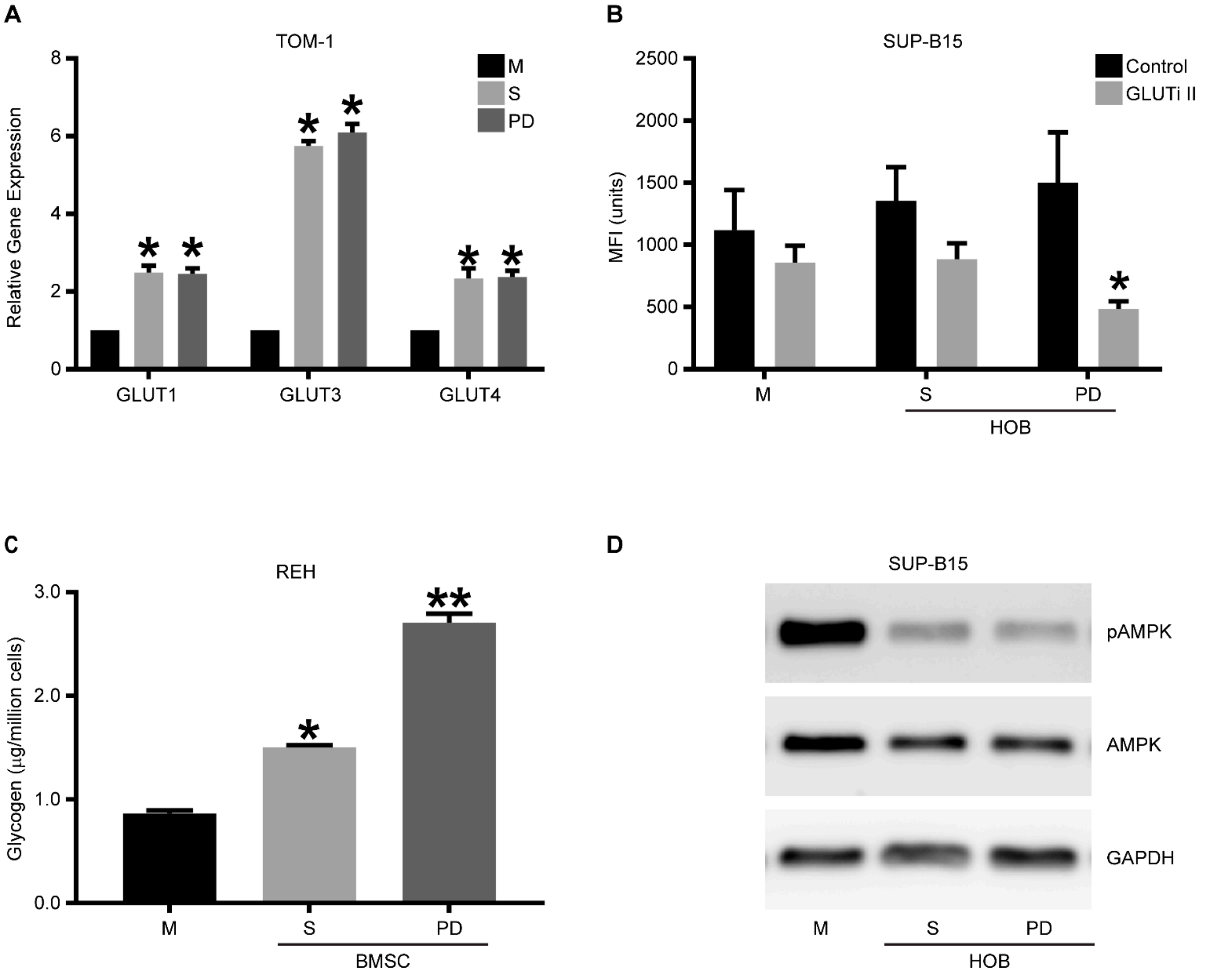

Figure S2. ALL cells are characterized by anabolic metabolism in co-culture. ALL cells were co-cultured with HOB or BMSC, and the cells in suspension (S) and the phase dim (PD) cells were isolated and compared to ALL cells grown in media alone (M). **(A)** RT-PCR was used to determine the expression levels of GLUT-1, 3, and 4. Data is represented as mean ± SEM and is a representative of an experiment performed in triplicate at least two independent times. **(B)** The different cell populations were treated with a pan-GLUT inhibitor (GLUTi II) or vehicle control followed by incubation with 2-NBDG. Flow cytometry was used to measure glucose content in cells shown as mean fluorescent intensity (MFI). **(C)** A glycogen colorimetric assay was used to measure the glycogen content in the isolated cell populations from BMSC co-culture. **(D)** Western blot analysis was used to measure the amount of AMPK and phospho-AMPK. The blots are representative of an experiment performed three independent times. * $p<0.05$ when compared to cells grown in media alone. ** $p<0.05$ when compared to cells grown in media alone or suspension.

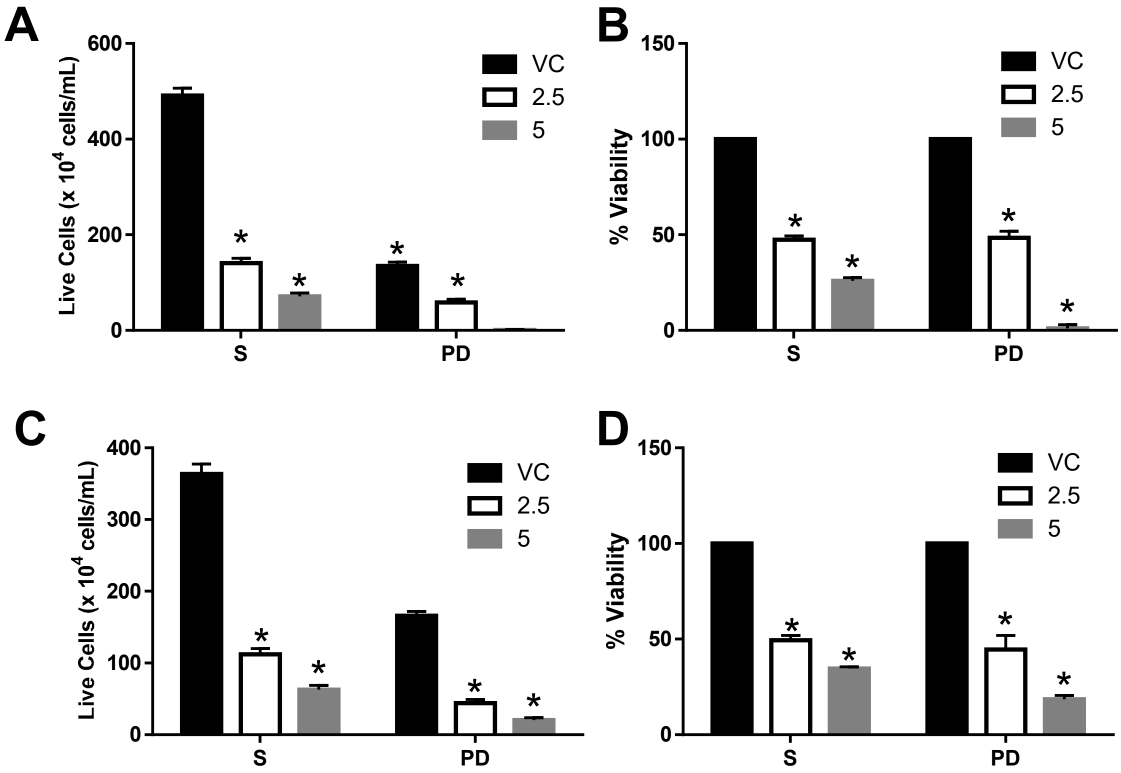

Figure S3. Metabolic targeted GLUTi II inhibitor decreases viability in ALL cells in co-culture. ALL cells were co-cultured with HOB and treated with GLUTi II pan-GLUT inhibitor, either at 2.5 µM or 5 µM. Following treatment, the cells in suspension (S) and the phase dim (PD) cells were isolated to determine cell viability. **(A)** Number of live REH cells. Data is represented as mean ± SD; N = 3 (**B)** Percent viability of REH cells. Data is represented as mean ± SD; N = 3 (**C)** Number of live SUP-B15 cells. Data is represented as mean ± SD; N = 3 (**D**). Percent viability of SUP-B15 cells. Data is represented as mean ± SD; (N = 3) * p<0.05 when compared to cells grown in media alone. ** p<0.05 when compared to cells grown in media alone or suspension. VC= vehicle control

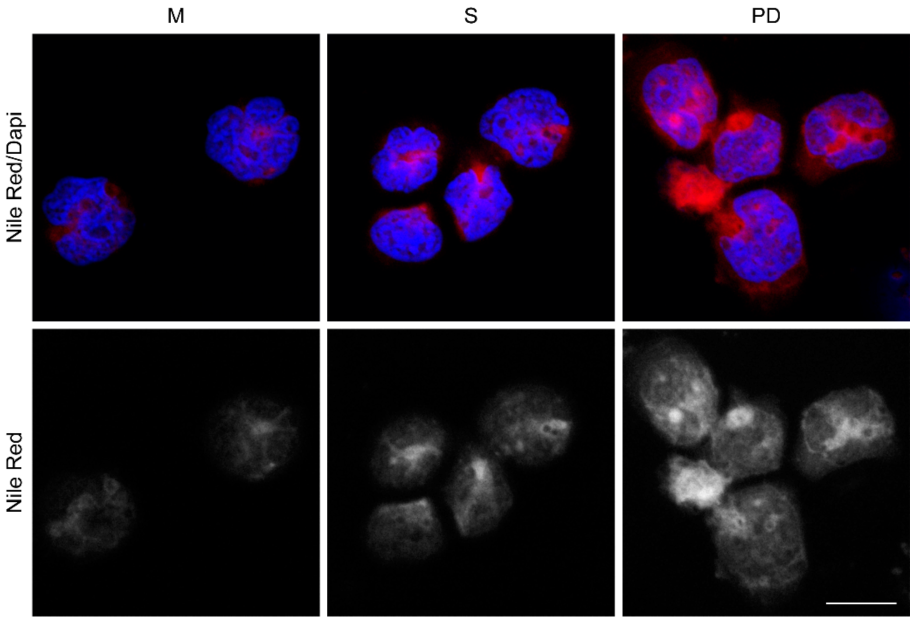

Figure S4. Drug-resistant phase dim (PD) cells have increased lipid content. TOM-1 cells were co-cultured with HOB, and the cells in suspension (S) and the phase dim (PD) cells were isolated and compared to TOM-1 cells grown in media alone (M). Nile Red staining was completed to visualize lipid content. Scale bar = 10 μm.

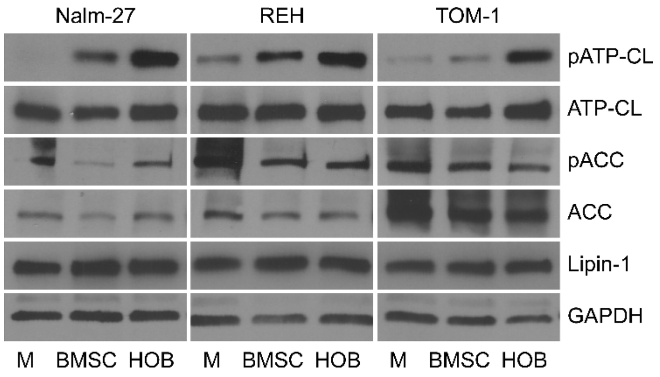

Figure S5. The fatty acid synthesis pathway is activated in ALL cells. ALL cells co-cultured with either BMSC or HOB were isolated and compared to ALL cells grown in media alone (M). The blots are representative of an experiment performed three independent times.

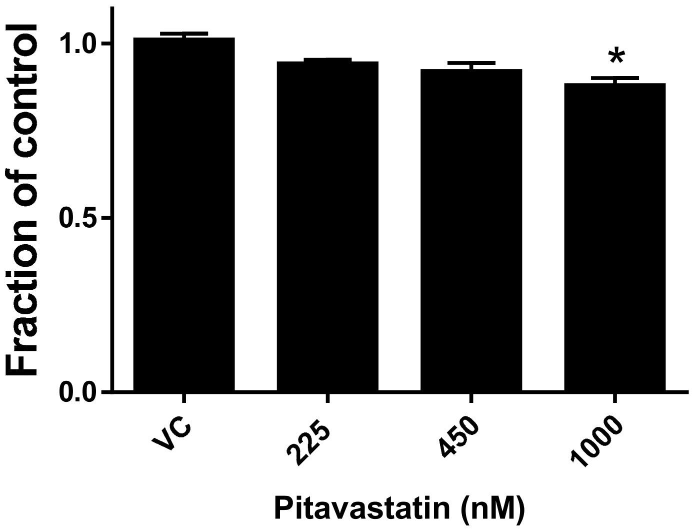

Figure S6. AML cells treated with pitavastatin. Cells were treated for 48 hours with pitavastatin, and cell proliferation measured with the WST-8 assay kit. Each bar represents avg + S.E., where N = 3, repeated three times. Statistical significance *P<0.05 .
